# Tunable Neuromorphic Switching Dynamics via Porosity Control in Mesoporous Silica Diffusive Memristors

**DOI:** 10.1021/acsami.3c19020

**Published:** 2024-03-18

**Authors:** Tongjun Zhang, Li Shao, Ayoub Jaafar, Ioannis Zeimpekis, Cornelis H. de Groot, Philip N. Bartlett, Andrew L. Hector, Ruomeng Huang

**Affiliations:** † School of Electronics and Computer Science, University of Southampton, Southampton SO17 1BJ, United Kingdom; ‡ School of Chemistry, University of Southampton, Southampton SO17 1BJ, United Kingdom

**Keywords:** mesoporous silica, diffusive memristors, neuromorphic switching, short-term memory, ion dynamics

## Abstract

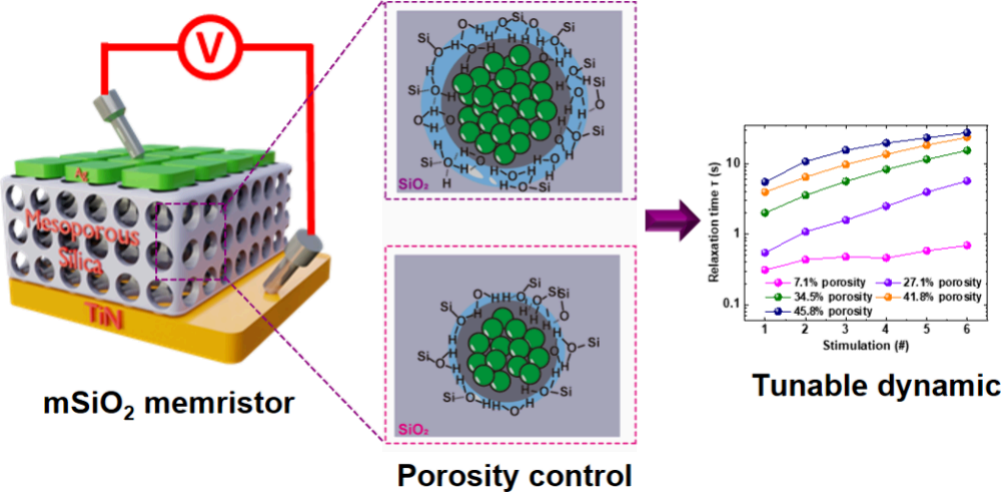

In response to the growing need for efficient processing of temporal information, neuromorphic computing systems are placing increased emphasis on the switching dynamics of memristors. While the switching dynamics can be regulated by the properties of input signals, the ability of controlling it via electrolyte properties of a memristor is essential to further enrich the switching states and improve data processing capability. This study presents the synthesis of mesoporous silica (mSiO_2_) films using a sol–gel process, which enables the creation of films with controllable porosities. These films can serve as electrolyte layers in the diffusive memristors and lead to tunable neuromorphic switching dynamics. The mSiO_2_ memristors demonstrate short-term plasticity, which is essential for temporal signal processing. As porosity increases, discernible changes in operating currents, facilitation ratios, and relaxation times are observed. The underlying mechanism of such systematic control was investigated and attributed to the modulation of hydrogen-bonded networks within the porous structure of the silica layer, which significantly influences both anodic oxidation and ion migration processes during switching events. The result of this work presents mesoporous silica as a unique platform for precise control of neuromorphic switching dynamics in diffusive memristors.

## 1 Introduction

The rapid expansion of big data and the increasing adoption of cloud computing applications have prompted the exploration of advanced information processing paradigms.^1^ While the conventional von Neumann computing architecture excels in computational capabilities, it is plagued by its constraint in data transfer between the physical computation and memory units, resulting in increased energy consumption and operational inefficiencies.^2,3^ Driven by the quest for energy- and time-efficient computation, neuromorphic computing has emerged as a transformative technology, marking a significant departure from the traditional von Neumann architecture. The human brain excels in processing intricate information with exceptional efficiency and minimal energy consumption, thanks to its integration of data processing and storage within the same neuronal and synaptic units.^4,5^ Neuromorphic computing aspires to replicate these extraordinary traits by developing novel electronic or optical devices that can emulate the behavior of biological neurons and synapses to achieve brain-like in-memory computing.^2,6^

Diffusive memristors stand out as prime candidates for neuromorphic computing among various bioinspired devices. A diffusive memristor is a two-terminal device whose switching is governed by the fast movement of diffusive species (e.g., Ag, Cu). In response to an electrical stimulus, the internal distribution of ions is disrupted, which leads to changes in the overall device resistance. As a device with similar physical behavior to the biological Ca^2+^ dynamics in pre/post-synaptic compartments, a diffusive memristor has the potential to faithfully emulate the synaptic function and enable broad applications in neuromorphic computing. Depending on the application, memristors are required to show different neuromorphic plasticity. For nontemporal or static information processing, memristors with nonvolatility and long-term plasticity (LTP) are required to construct the feedforward neural networks (FNNs). Several memristors have been developed to show such behavior and perform tasks such as pattern recognition and classification with high accuracy and low power consumption.^4,7−13^

Temporal signal processing is another important subset in modern data analysis and interpretation, given that a substantial portion of the data we encounter nowadays are inherently time-dependent. For example, the identification of irregular patterns and trends in a patient’s vital signs (e.g., heart rate, blood pressure) in real-time can signal health issues and contribute to accurate and timely diagnosis;^11,13,14^ analysis of the temporal data on temperature, humidity, and wind speed allows meteorologists to make weather predictions and assess long-term climate trends.^13,15−17^ Tasks that deal with temporal information demand memristors that show volatility and short-term plasticity (STP) for recurrent neural networks (RNNs).^18^ Recently, several pioneering studies have been reported that implement memristors with such behavior into physical reservoir computing (RC) systems, an advanced RNN configuration, to showcase the capability of processing temporal information.^9,10,13,15,16,19−25^ Du et al. developed an RC system based on WO_*x*_ memristor arrays to solve a second-order nonlinear dynamic task;^26^ Zhong et al. adopted the TiO_*x*_-based memristor in the RC system and realized superior temporal computational performance in tasks such as time-series prediction, temporal arrhythmia detection, and spatiotemporal dynamic gesture recognition.^13^

Crucially, for temporal signal processing, different computational tasks demand different temporal properties from memristors. The temporal requirements for speech recognition, where fast computing is required in a short period, are likely to be different to those of IoT applications where the signals from sensor networks arrive at certain intervals.^18^ The temporal properties in diffusive memristors depend largely on the ion dynamics in the active layers of the memristor.^21,27,28^ For conventional dense films, ions will travel through the grain boundaries and defective sites to form conductive filaments.^29−31^ The structural engineering of memristors plays a critical role in governing and tuning their temporal responses to fulfill the computing system’s application requirements.^27,32,33^ A few previous works have explored the possibilities of achieving effective reconfigurability through alterations in the material structure under given temporal signals.^3,24,34−36^ However, precise control over ion dynamics in dense films can be challenging due to the stochastic grain boundaries. Systematic control over the memristor dynamics via structure control is, therefore, still lacking.

Mesoporous nanocomposite materials feature fast electron transport and a high surface-to-volume ratio, conferring superior properties as the active layer in diffusive memristors.^37−41^ The porosity of mesoporous silica can be precisely engineered, and the synthesis process is straightforward.^41,42^ More importantly, the introduction of a porous structure into the electrolyte layer enables precise regulation of the ion dynamics through the control of pore geometry, providing the nano/atomic-scale control needed for diffusive memristors.^5,42,43^ Li et al. demonstrated short-term plasticity in an artificial memristive device using the different orientations of mesopores silica (mSiO_2_).^39^ Our previous work accomplished a physical reservoir computing system utilizing a 3D-structured, highly ordered mSiO_2_-based memristor.^44^ These works provide the possibility of using mesoporous silica as the active layer with a straightforward fabrication method.

In this work, we present tunable neuromorphic behaviors in highly ordered mesoporous silica-based memristors with controllable porous structures. The porosity of the devices is achieved by controlling the surfactant concentration in the solution. The as-fabricated devices exhibit both digital and analog switching behavior under different operating voltages. The resistive switching is modified by adjusting the architecture of Ag filaments, where the different porosities play an important role in the drift and diffusivity of Ag ions. By using these porosity structures, the STP behavior of our memristor, including both pulsed-induced current potentiation and relaxation, can be precisely controlled.

## 2 Experimental Section

### 2.1 Chemical Precursors

Triblock copolymer Pluronic F127 (*M*_w_ = 12600, PEO106–PPO70–PEO106), tetraethyl orthosilicate (TEOS), and 37% hydrochloric acid (HCl) were purchased from Sigma-Aldrich Company Ltd. No further purification was required in the fabrication. 1 M HCl was diluted from 37% HCl with deionized water. Ethanol (≥99.8% concentration) and dichloromethane (DCM) were acquired from Fisher Scientific.

### 2.2 Synthesis of Mesoporous Silica Films

The precursor solution was prepared based on the evaporation-induced self-assembly method. 1.0 g of TEOS was prehydrolyzed by dissolving in 5.64 g of ethanol, 0.80 g of deionized water, and 0.10 g of 1 M hydrochloric acid and stirring at 338 K for 45 min. Various amounts of triblock copolymer F127 (0.0605, 0.1814, 0.3024, 0.4234, or 0.5443 g) were dissolved in 5.64 g of ethanol under ambient conditions. The two solutions were mixed and stirred at room temperature for 60 min. The final molar ratios of the precursor solution were as follows: TEOS:F127:HCl:H_2_O:EtOH = 1:(0.001, 0.003, 0.005, 0.007, or 0.009):0.021:9.2:51.

Dip-coating was used to coat the silica films onto the TiN bottom electrodes. The cleaned TiN substrates were vertically immersed into the prepared precursor solution and withdrawn at the rate of 100 mm/min in a humidity chamber with 55% relative humidity at 298 K. After keeping in the humidity chamber (RH = 55%) for 72 h, the as-made films were aged at 120 °C for 10 h. The surfactant was removed by immersing in the DCM solution for 4 h and calcinated at 350 °C for 5 h afterward, after which they were referred to mesoporous silica films.

### 2.3 Device Fabrication

The Si/SiO_2_ substrates were 20 × 20 mm. A 200 nm-thick TiN film was reactively sputtered onto the Si/SiO_2_ substrate to form the bottom electrode using a Leybold Helios Pro XL Sputterer. The reactive sputtering process was performed with 3 kW of RF power using a Ti target in a nitrogen atmosphere. After mesoporous silica thin film deposition, 200 nm thick Ag top electrodes were deposited by e-beam evaporation (Leybold Lab 700) via a designed shadow mask containing the dimension gradient from 50 to 250 μm.

### 2.4 Characterization

GISAXS patterns were obtained from Rigaku SmartLab with a Hypix-3000 Detector System with a wavelength λ of 1.54 Å at an incident angle of 0.3°. Ellipsometry measurements were obtained from a Woollam M-2000 XI spectroscopic ellipsometer. Refractive index values were modeled using the Cauchy dispersion model. X-ray photoelectron spectroscopy measurements were performed using a Theta Probe System with an Al Kα source (photon energy = 1486.6 eV). All the binding energies were calibrated with respect to the peak of the adventitious C–C peak at 284.8 eV. Electrical properties of all memristors were performed at room temperature using a Keysight B1500 semiconductor characterization system connected to a probe station.

## 3 Results

**Figure 1:**
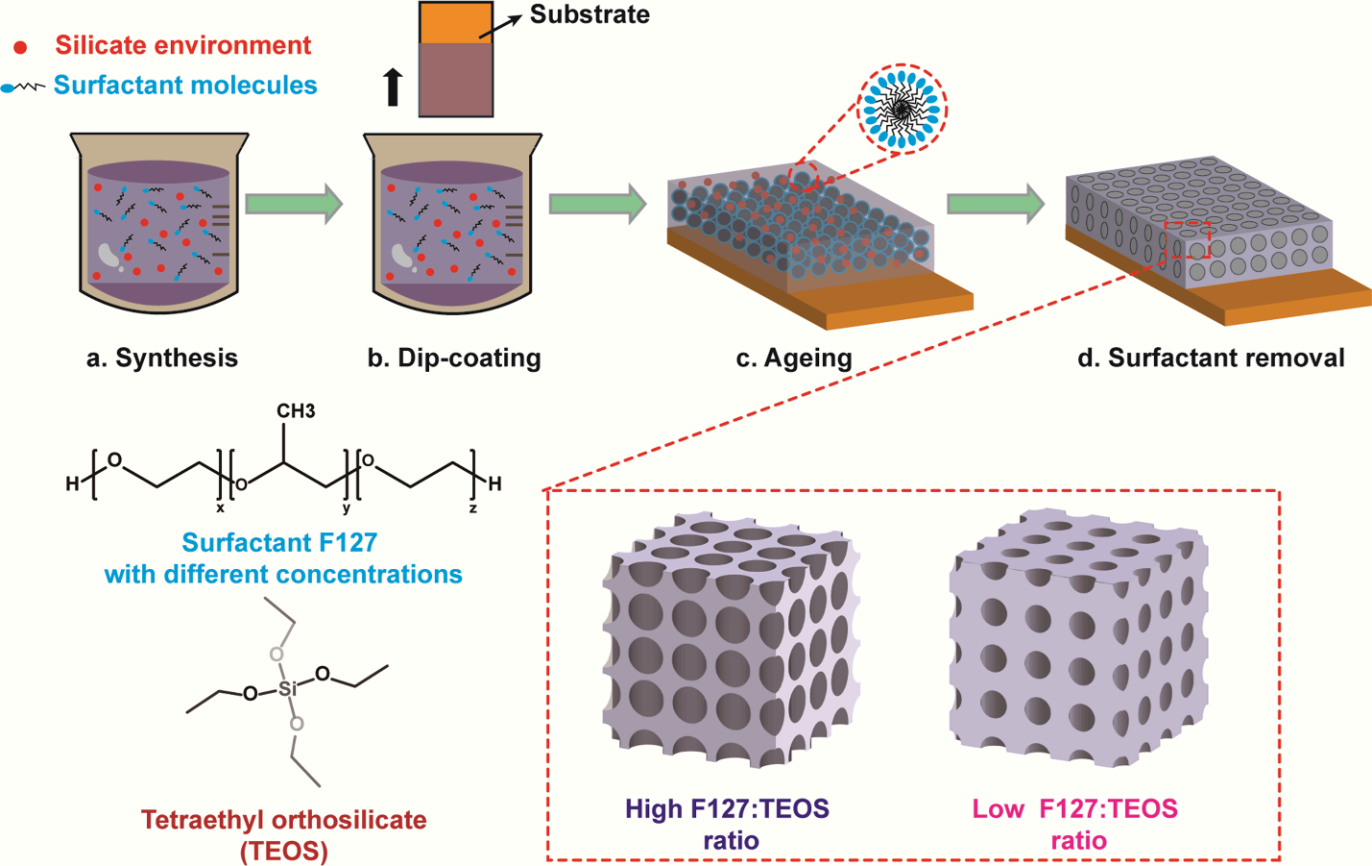
Schematic of the sol–gel process for mesoporous silica deposition. (a) TEOS and the triblock copolymer F127 surfactant are mixed in the solution of ethanol and hydrochloric acid in deionized water. (b) Dip-coating process to deposit the silica film onto a substrate. (c) Aging process that allows the completion of the evaporation process. (d) Annealing process that removes the micelles, leaving mesopores in the silica film.

A self-assembly sol–gel method was used to synthesize the silica film with the porous structure, as illustrated in Figure 1. The precursor solution was prepared by mixing the tetraethyl orthosilicate (TEOS), Pluronic F127 surfactant, ethanol, and hydrochloric acid in deionized water (Figure 1a). A dip-coating process was adopted to deposit the silica films (Figure 1b). After dipping the substrate in the precursor solution and then withdrawing it, evaporation occured at the air/film surface. When the concentration of the surfactant was above the critical micelle concentration, micelles started to form by the aggregation of the hydrophobic tails of the surfactant. With time, TEOS molecules hydrolyzed and condensed around the ordered arrays of surfactant micelles and the mesostructured hybrid network were formed (Figure 1c). After the surfactants were removed, the mesoporous silica films were obtained (Figure 1d). The porosity of the film was controlled by varying the ratio of F127 to TEOS from 0.001 to 0.009 (details in the Experimental Section).

**Figure 2:**
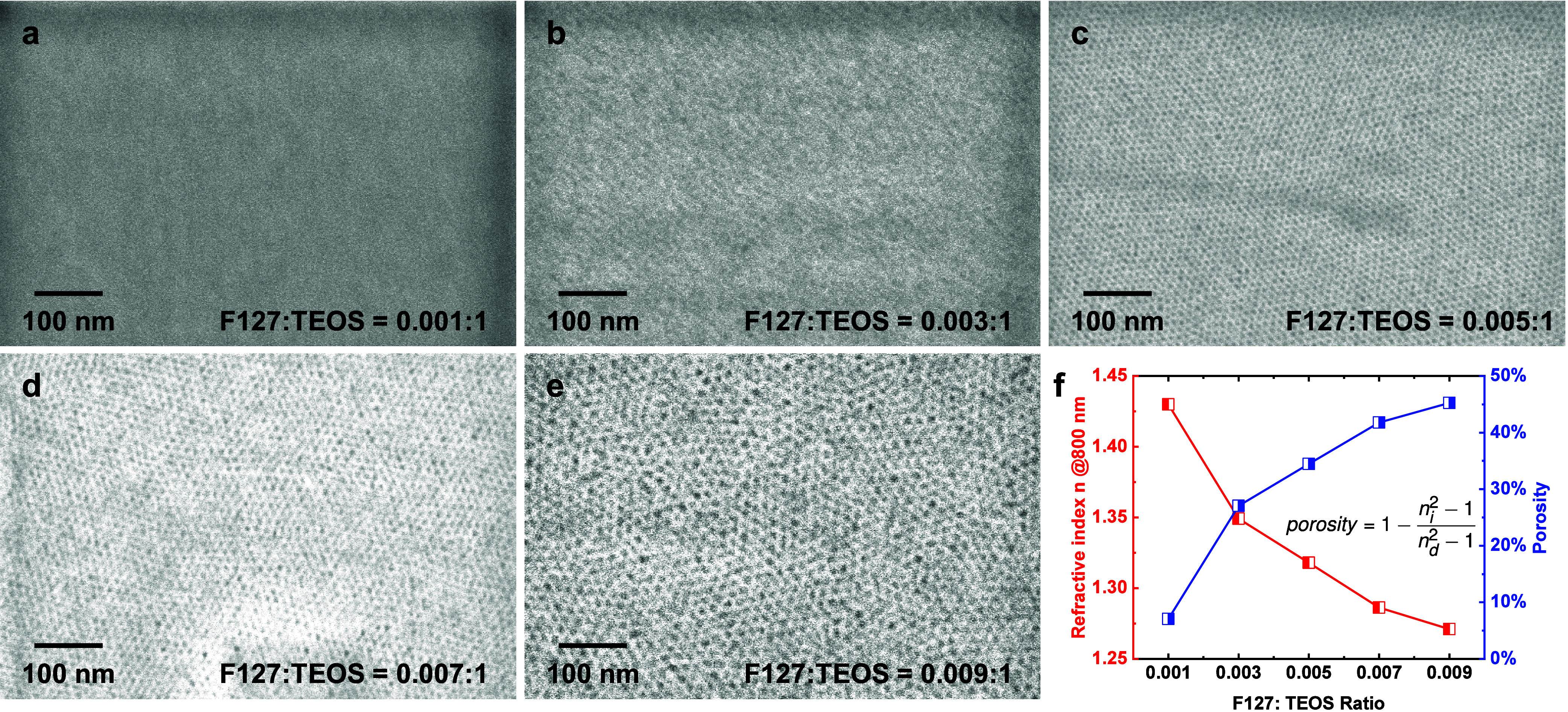
SEM images showing the pore structure of the samples prepared with different F127:TEOS ratios: (a) 0.001 F127:1 TEOS ratio; (b) 0.003 F127:1 TEOS ratio; (c) 0.005 F127:1 TEOS ratio; (d) 0.007 F127:1 TEOS ratio; (e) 0.009 F127:1 TEOS ratio; (f) refractive index and calculated porosity change of mesoporous silica films prepared with different F127:TEOS ratios.

The as-deposited mesoporous silica films were characterized by high-resolution scanning electron microscopy (SEM) as shown in Figure 2. At a low surfactant ratio, the pores are barely visible (Figure 2a). This is unsurprising as the nanoscale micelles assembled under such conditions could be too small. However, with an increasing F127:TEOS ratio, larger micelle structures are formed, leaving larger pores in the film. This is observed in the following SEM images where a higher surfactant concentration results in larger, and more visible nanopores in the film (Figure 2b–e). Despite having different pore sizes, all of the pores are uniformly distributed in a given film. The highly ordered three-dimensional structures of these mesoporous films were characterized by grazing-incidence small-angle X-ray scattering (GISAXS) and are shown in Figure S1 in the Supporting Information. To establish the porosity of the silica films produced with different surfactant ratios, we determined the refractive indices of those films using ellipsometry. The obtained ellipsometry results were fitted with the Cauchy dispersion model to derive the refractive indices and thicknesses of the films (shown in Figure S2). Figure 2f plots the refractive indices at 800 nm as a function of the surfactant ratio. The decrease in the refractive index from 1.43 to 1.27 at 800 nm implies more air in the film due to increasing film porosity. The actual porosity value can be evaluated by comparing the refractive indices of the porous silica film with that of the thermally grown one using the following equation:
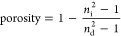
1where *n*_i_ is the refractive index of the porous silica film and *n*_d_ is the refractive index of the thermal-grown SiO_2_ layer.^45^ As shown in Figure 2f, the calculated porosity increases from 7.1% at the low F127:TEOS ratio to 45.3% at the high F127:TEOS ratio. This is consistent with what we observed in the SEM images. Atomic force microscopy (AFM) topography images (shown in Figure S3) indicate the smoothness of all films with average surface roughness less than 1 nm. These results confirm that the porosity of our flat mesoporous film can be controlled systematically via the dip-coating deposition process.

**Figure 3:**
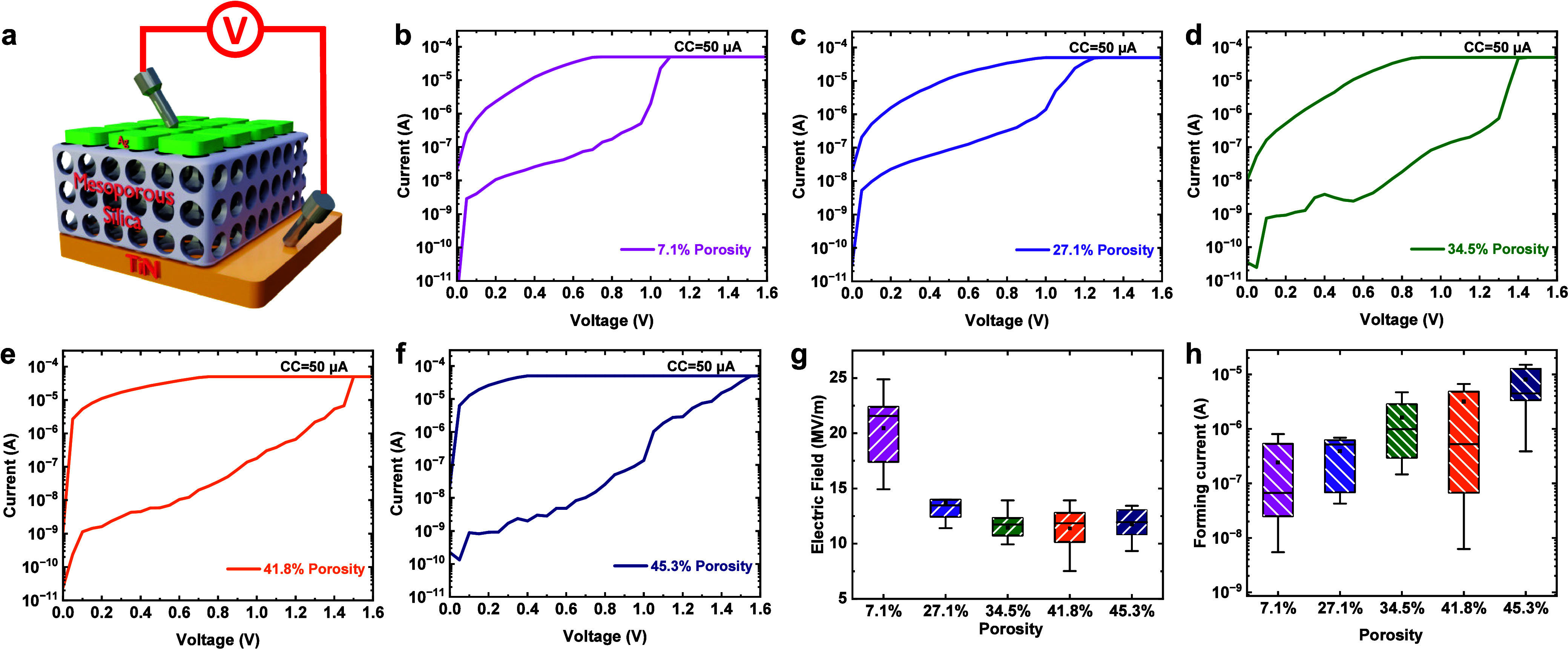
Resistive switching behavior of mesoporous silica memristors: (a) schematic of the mSiO_2_-based memristor. (b–f) *I*–*V* curve showing the forming process for different porosity mesoporous silica devices. (g) Forming electric field and (h) formation current (measured @ 0.1 V) distribution for mSiO_2_ memristors with different porosities.

We now focus on the impact that different porosities have on the electrical switching properties of the silica films. Memristor devices were fabricated by sandwiching silica-thin films with different porosities between active Ag top electrodes and inert TiN bottom electrodes. The schematic diagram of the device structure is illustrated in Figure 3a. The detailed fabrication process is provided in the Experimental Section. All five types of memristor show high resistance in their pristine state, and electroforming steps were required to initialize them as shown in Figure 3b–3f. This was achieved by applying a positive DC bias from 0 to +2 V on the Ag top electrode while keeping the TiN bottom electrode grounded. A predefined compliance current (CC) of 50 μA was required to prevent the device from breaking down, and the forming voltage is defined as the voltage when the CC is reached. Figure 3g presents the forming electrical field (voltage/film thickness) of each sample. It can be observed that films with higher porosity require lower electric fields to form a conductive channel. We have previously shown that the current conduction in our mesoporous silica memristor is achieved by the formation and rupture of Ag filaments in the pore matrix.^44^ The reduction in the electroforming electrical field suggests that the higher porosity facilitates ion movement in the silica film. Concurrently, the forming currents correlate with increasing porosity (shown in Figure 3h). This implies that stronger conductive filament(s) were established in films with higher porosities after the electroforming process. More discussion of this will be given later in the Discussion section.

**Figure 4:**
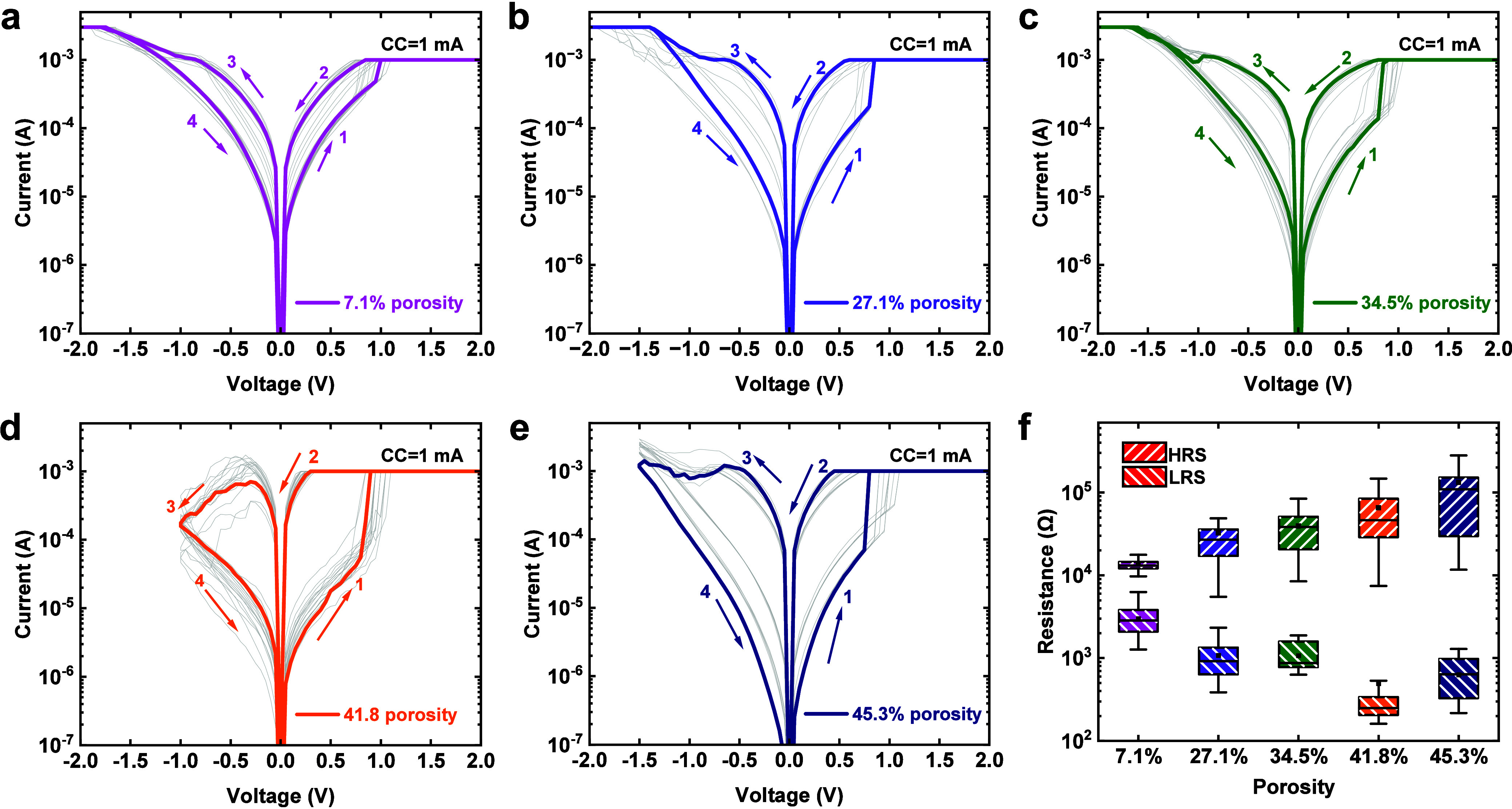
*I*–*V* characteristics showing the typical nonvolatile resistive switching properties of the mSiO_2_ memristors under a CC of 1 mA. Porosities of (a) 7.1, (b) 27.1, (c) 34.5, (d) 41.8, and (e) 45.3%. (f) Resistance state distribution of nonvolatile resistive switching behaviors.

After the electroforming process, the mesoporous silica memristors can be switched in a nonvolatile switching style with a voltage of 2 V and a high CC of 1 mA. As shown in Figure 4a–4e, when the positive DC sweep is applied, the memristor can be switched into a low resistance state (LRS) after an abrupt current increase. A negative bias is then required to reset the device back to a high resistance state (HRS), showing typical bipolar switching behavior. Memristors based on all five silica films with different porosities show similar switching characteristics with good retention and DC endurance (shown in Figure S4 and S5, respectively). Figure 4f plots the resistance states of all memristors as a function of the film porosity. It can be observed that, despite similar switching behavior, films with higher porosity give rise to lower LRS and higher HRS, resulting in a larger ON/OFF ratio from ca. 10 to over 100. This further suggests that stronger conductive filaments are formed and switched in the films with high porosities. Interestingly, the formation and rupture of these stronger filaments do not seem to require larger switching voltages (shown in Figure S6), implying that ion movement is favored in these films. To elucidate the conduction mechanisms for both ON and OFF states, we replot all *I*–*V* curves on a log–log scale, as shown in Figure S7. All memristors demonstrate a similar conduction mechanism where the current in HRS fits well with the space-charge-limited-current (SCLC) model, and the LRS is governed by ohmic conduction (details in the Supporting Information).

**Figure 5:**
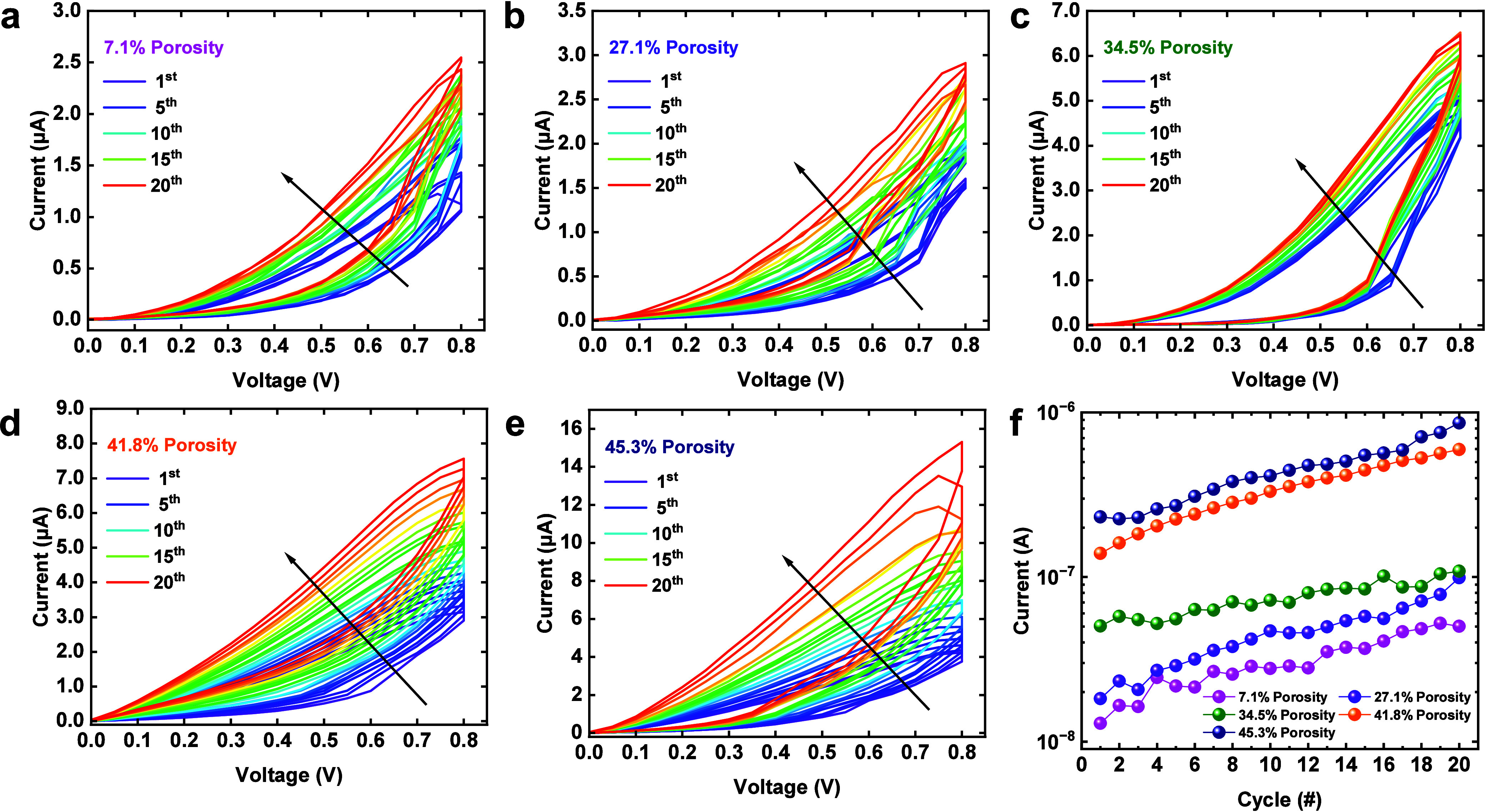
Consecutive switching *I*–*V* characteristics of samples with different porosities showing the analogue switching behavior. Porosities of (a) 7.1, (b) 27.1, (c) 34.5, (d) 41.8, and (e) 45.3%. (f) Current changes versus consecutive cycles of the LRS of the device. The current values were read at 0.1 V.

A key requirement for memristors in neuromorphic computing applications is the analog switching behavior, which arises from the capability of gradual modulation of the conductance. Figure 5a–e present the current responses of the memristors under 20 consecutive DC sweeps. In all cases, gradual conductance modulation can be achieved upon continuous electrical stimulation between 0 and 0.8 V. Figure 5f summarizes the current obtained as a function of the number of DC sweep cycles (measured at 0.1 V). The results show that the current state increases gradually with each repeating DC cycle in all devices, indicating the analog switching behavior of our mSiO_2_-based memristors. The HRS of our memristor exhibits a similar trend, wherein the current states progressively increase with an increase in cycles (shown in Figure S8). Moreover, the current modulation level can be controlled by the porosity of silica layers over 1 order of magnitude with higher porosities resulting in larger current levels. It is worth noting that the conductance prior to each stimulus is lower than that after previous stimuli. This implies the spontaneous dissolution of conductive filaments between two consecutive DC sweeps with an interval of 1 s. Such spontaneous current decay is indicative of the STP in our mSiO_2_-based memristors.

**Figure 6:**
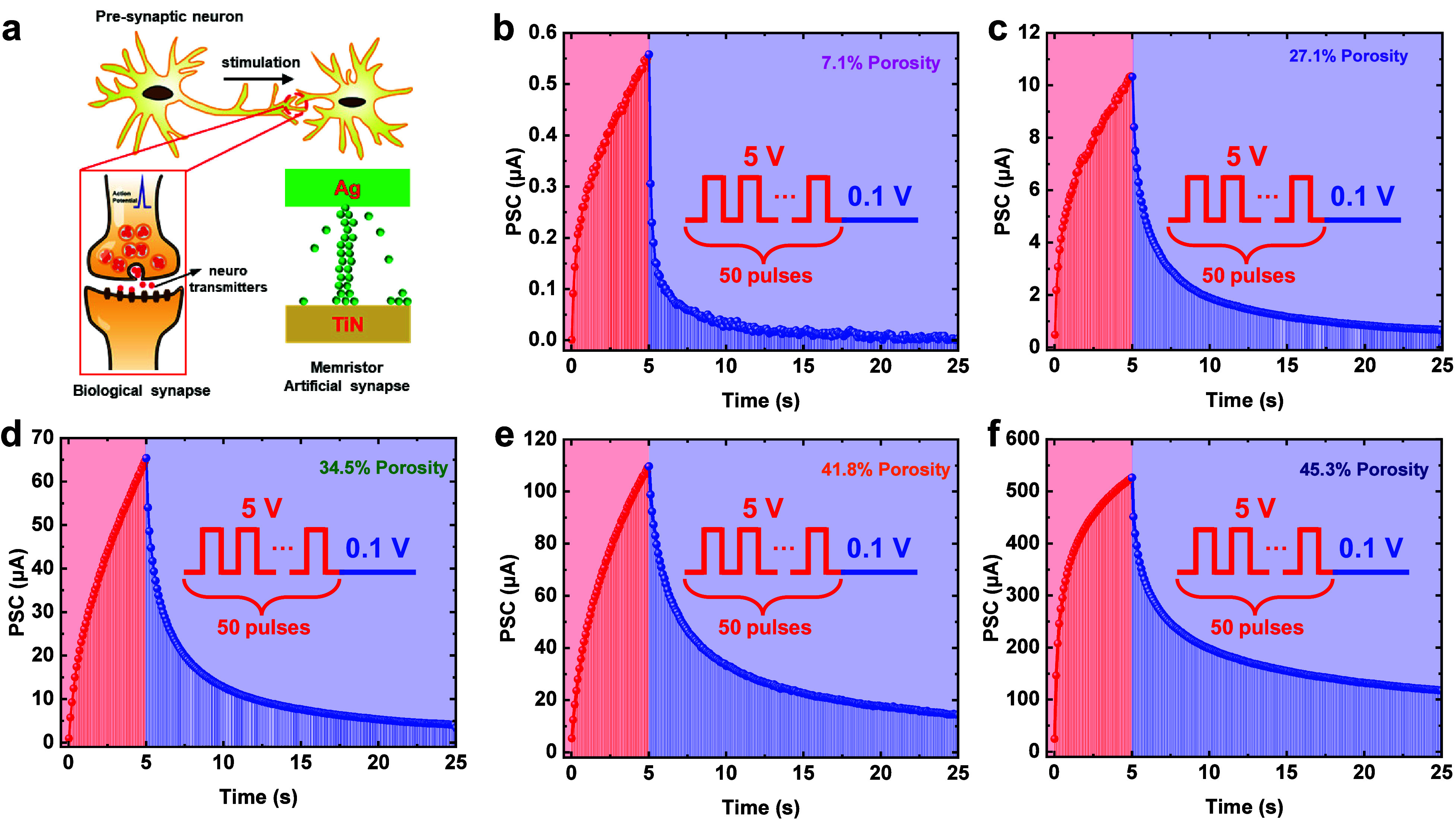
(a) Schematic representation of a biological neural network and a memristor device showing the correspondence between biological and electronic synapses; gradual PSC change with a series of voltage pulses (+1 V, 50 ms duration) and the subsequent autodecay showing STP behavior on the device with 45.3% porosity: (b) 7.1, (c) 27.1, (d) 34.5, (e) 41.8, and (f) 45.3% porosities.

Neurons and synapses constitute the fundamental components of natural neural systems.^4,46,47^ In the neural system, the information transfer relies on the synapse structure between two neurons (pre- and postsynaptic neurons) as depicted in Figure 6a. Neurobiologically, synaptic weights can be changed in response to external stimulations. This is achieved via the release of neurotransmitters (i.e., Ca^2+^), which travel to the synaptic cleft, the narrow space between pre- and postsynaptic neurons, after receiving the stimulation. The synaptic connection strength between pre- and postsynaptic neurons is therefore dynamically modulated.^48^ After the stimulation is removed, the neurotransmitter concentration returns to the baseline level with an associated synaptic weight. The process normally occurs over milliseconds to minutes and manifests itself as typical STP behavior. Such behavior serves as the biological basis for human brain information processing and memorizing,^4,49,50^ and allows synapses to perform critical computational functions in neural circuits.^47^ Similarly, memristor devices can emulate the characteristics of biological synapses (shown in the top right sketch of Figure 6a). Under electrical stimulation, the conductance of memristors can be modified, showing current enhancement characteristics. When stimulation is removed, a constant relaxation process is obtained with temporal constraints.^49^ Our mesoporous silica memristor can emulate this STP behavior by modulating its conductance through the formation and spontaneous dissolution of Ag filaments in the porous matrix. The drift and diffusion of the Ag^+^ ions in the silica layer mimic the generation and restoration of neurotransmitter ion concentration differentials in biological synapses.^44^Figure 6b–f exhibits the electrical responses of our memristors upon consecutive stimuli. The stimulation pulse consists of a series of 50 pulses with a 50 ms duration and 50 ms interval. The synaptic weight, which is defined as the postsynaptic current (PSC), is collected at a read voltage of 0.1 V for memristors with different porosities. It can be observed that all devices show a similar trend, where the currents gradually increase upon the application of electrical pulses. However, the current level varies with the porosity of the silica film, where a large porosity leads to a higher PSC level. Following the withdrawal of stimulation, the PSC decays into its initial current state automatically. It is confirmed that short-term plasticity behaviors can be obtained in all samples with different porosities, which is crucial for application in spatiotemporal information processing.

**Figure 7:**
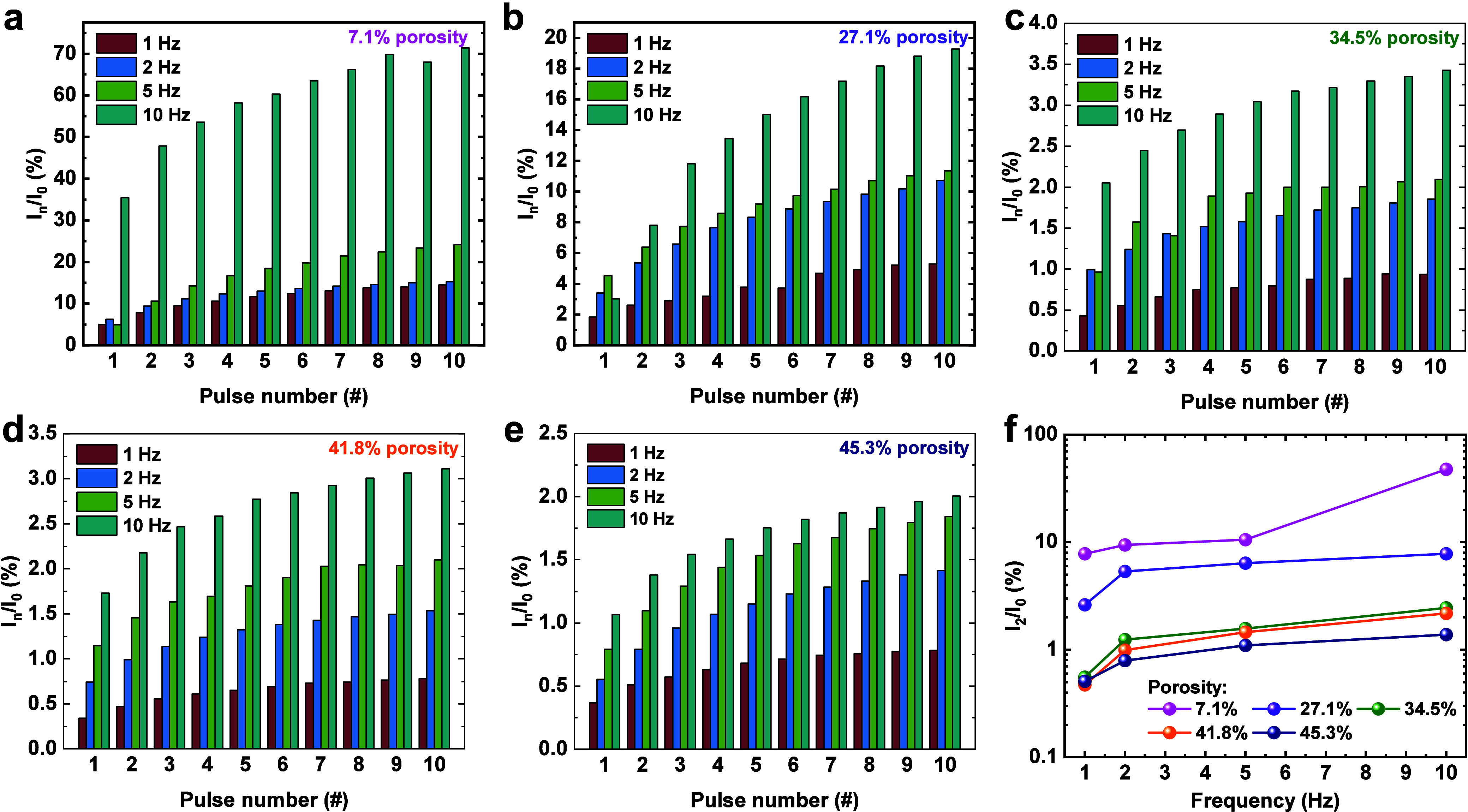
Gradual PSC current change showing SRDP behavior: among samples with different porosities: (a) 7.1, (b) 27.1, (c) 34.5, (d) 41.8, and (e) 45.3%. (f) The facilitation ratio concerning the spike frequency is determined after the second pulse.

To quantitatively analyze the dynamic response of the memristors with different porosities, a set of pulses with a fixed duration (50 ms) but varying intervals (frequencies) were applied to the memristors. The facilitation ratios were evaluated by the following equation:
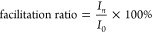
2where *I*_*n*_ is the PSC after pulse number *n* and *I*_0_ is the initial current value. Figure 7a–e exhibits the facilitation ratios of memristor devices after the application of four sets of electrical pulses with frequencies varying from 1 to 10 Hz. In all cases, the current facilitation increases with the number of pulse stimulations. For each porosity, the facilitation is more drastic with a higher pulse frequency, demonstrating the typical spike-rate-dependent plasticity (SRDP) behavior. Apart from the stimulations, the facilitation can also be controlled via the mSiO_2_ porosity. Compared with low porosity films, mSiO_2_ with high porosity demonstrates a significantly reduced PSC facilitation level despite the memristor operating at a high current level. The lowest PSC facilitation is obtained in the memristor with 45.3% porosity. Figure 7f presents the facilitation ratios after the second pulse as a function of the pulse frequency. An over 10-fold difference in the facilitation ratio can be observed for mSiO_2_ memristors with different porosities, suggesting that the mesostructured architecture can serve as a unique platform to develop artificial synapses with different current modulation properties.

**Figure 8:**
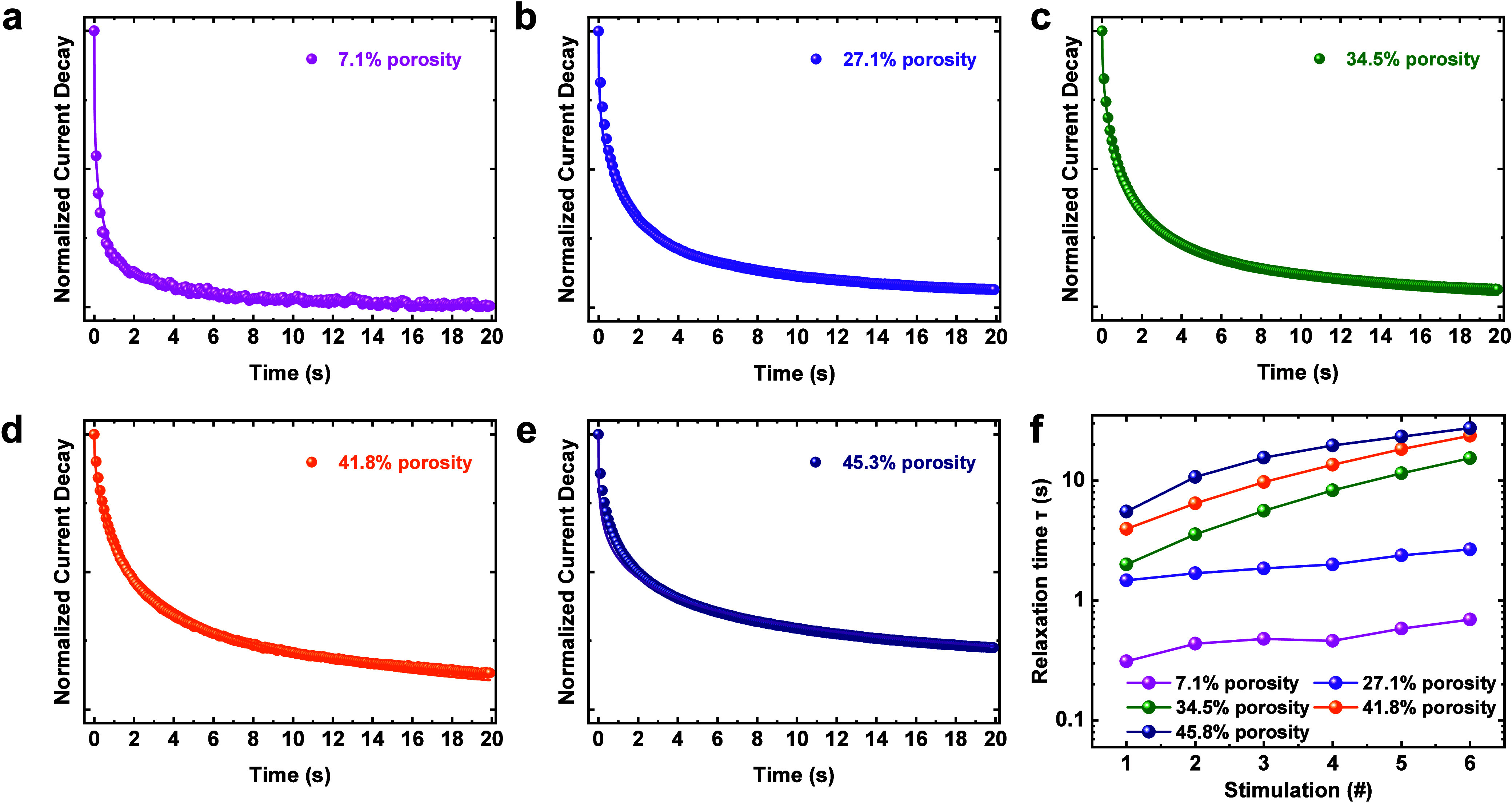
Retention loss data and fitting results of memristors with different porosities after the mentioned pulse trains. Porosities of (a) 7.1, (b) 27.1, (c) 34.5, (d) 41.8, and (e) 45.3%. (f) Fitted relaxation time contrast among five different samples in a series of six pulse trains.

In addition to the dynamic response of the PSC, the spontaneous current decay after the removal of pulses is also an important characteristic of memristors and crucial to their temporal signal processing capability. Six series of pulse trains, each containing 50 pulses (1 V potential, 50 ms duration, and 50 ms interval), were applied on our mSiO_2_ memristors with a 20 s gap between each pulse train. Their current responses are presented in Figure S8 where a series of potentiation and automatic relaxation are observed, showing typical learning-forgetting-rehearsal behavior. For quantitative comparison of the relaxation process, we fit the relaxation time of each current decay by adopting a stretched-exponential based function (SEF):
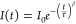
3Here, *I* is the relaxation current at a given time *t*, the prefactor *I*_0_ is the initial current at the start of decay (*t* = 0 s), τ is the characteristic relaxation time, and β is the stretch index, which was chosen to be between 0.3 and 0.5 for optimizing fitting results.^48^Figure 8a–f plots the fitting of current relaxation after the first series of pulse trains. It can be observed that the fitted relaxation time τ increases from 0.3 s for the memristor with a low porosity to 5.5 s for the one with a high porosity. The relaxation times for all memristors after each series of pulse trains are presented in Figure 8f. In all cases, longer relaxation times are obtained for memristors with a higher porosity. For each memristor, the relaxation increases with the number of pulse train series applied. These findings reveal that nanostructure modulation is an effective way to control the temporal dynamics of a memristor. Table S1 provides an overview of the memristive capabilities observed in different porous-based memristors where our devices exhibit distinctive pore control and management of relaxation time windows.

## 4 Discussion

**Figure 9:**
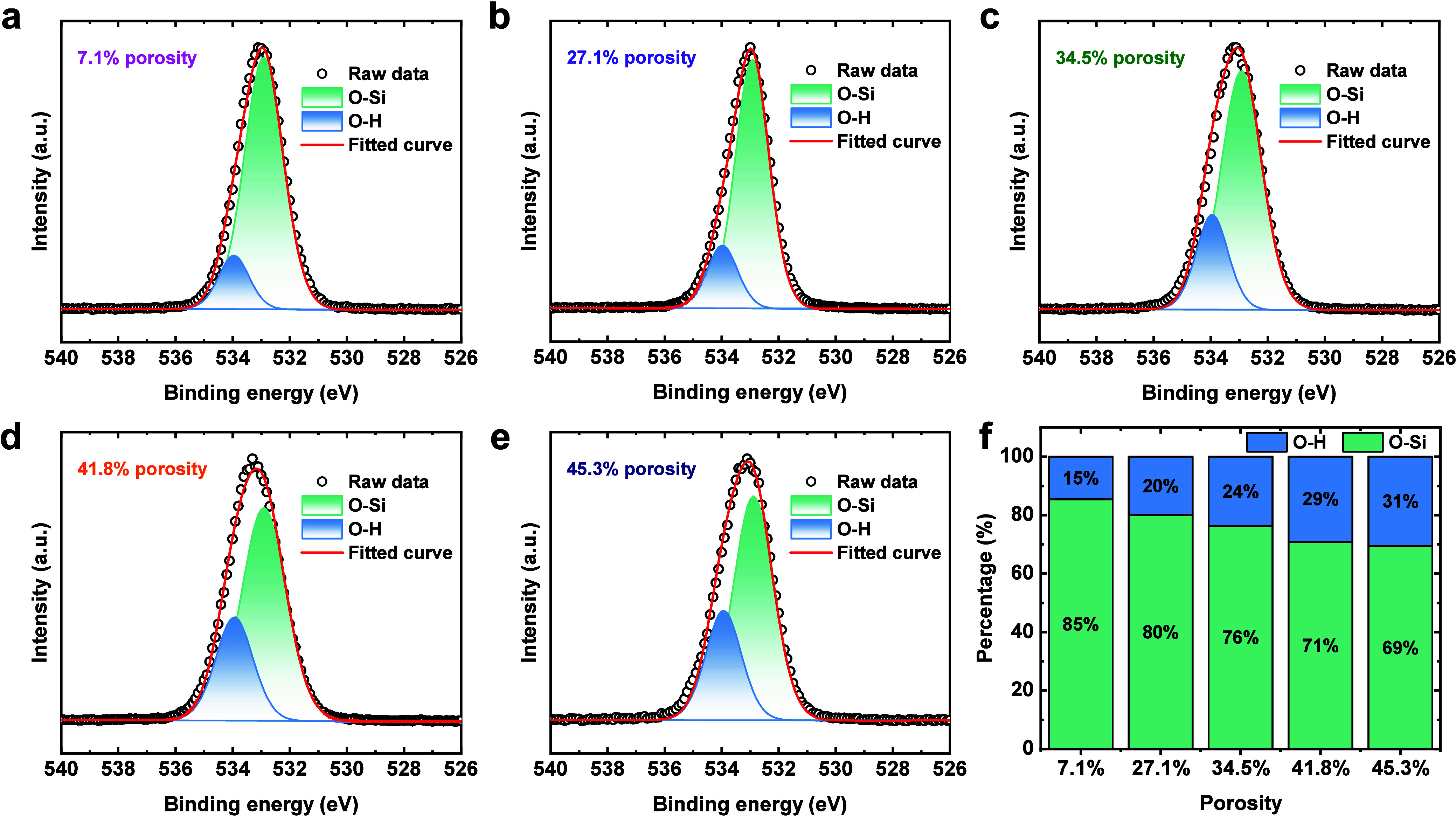
XPS profiles of the O 1s core level of the films with different porosities: (a) 7.1, (b) 27.1, (c) 34.5, (d) 41.8, and (e) 45.3%; (f) composition ratio of O–Si and O–H bonding in the O 1s XPS spectra.

Here, we discuss the underlying physical switching mechanism of our mSiO_2_ thin film memristor, as well as its impact on temporal performance. Our research has shown that the switching mechanism of the memristor is governed by the formation and rupture of Ag filaments within this pore matrix.^44^ A key property of the pore matrix is its large surface area, which will absorb moisture during ambient exposure, leading to the formation of a thin hydrogen-bonded network at the pore walls.^51^ X-ray photoelectron spectroscopy (XPS) measurements were performed to examine the surface chemical state of the deposited mSiO_2_ thin films. Figure 9 presents the O 1s core level of the films with different porosity. The envelope peak moves gradually toward higher energy with increasing film porosity (shown in Figure S10). Both O–Si (binding energy 532.9 eV) and O–H (binding energy 534.0 eV) groups can be observed in all five films, suggesting the existence of such hydrogen-bonded networks within our mSiO_2_ thin films. More importantly, the intensity of the hydroxyl bonding displays a significant enhancement with an increase in film porosity (shown in Figure 9f). This implies that a larger amount of water molecules is absorbed by SiO_2_ films with larger pores. This is unsurprising as more pore wall surface area is available in larger pores for the adsorption of moisture from the ambient environment.^52,53^ Such hydrogen-bonded networks can then play a crucial role in anodic oxidation as well as ion migration processes, significantly affecting the switching dynamics of our memristor.

In the electroforming process, when a positive bias voltage is applied to the Ag electrode, the active Ag atoms are oxidized into Ag ions at the anode interface:
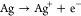
4

Concurrently, a counter-electrode reaction through the reduction of water molecules into OH^–^ ions will take place at the cathode interface to maintain electroneutrality:
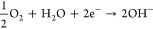
5

The presence of these water molecules in our mesoporous silica film strongly affects the ionization process of Ag at the top electrode. In particular, for mSiO_2_ films with high porosity, the redox reaction is significantly enhanced at both interfaces due to the large volume of water trapped in the nanopores. More Ag ions can therefore be generated and injected into the SiO_2_ matrix with a relatively low-forming electric field as shown in Figure 3.

**Figure 10:**
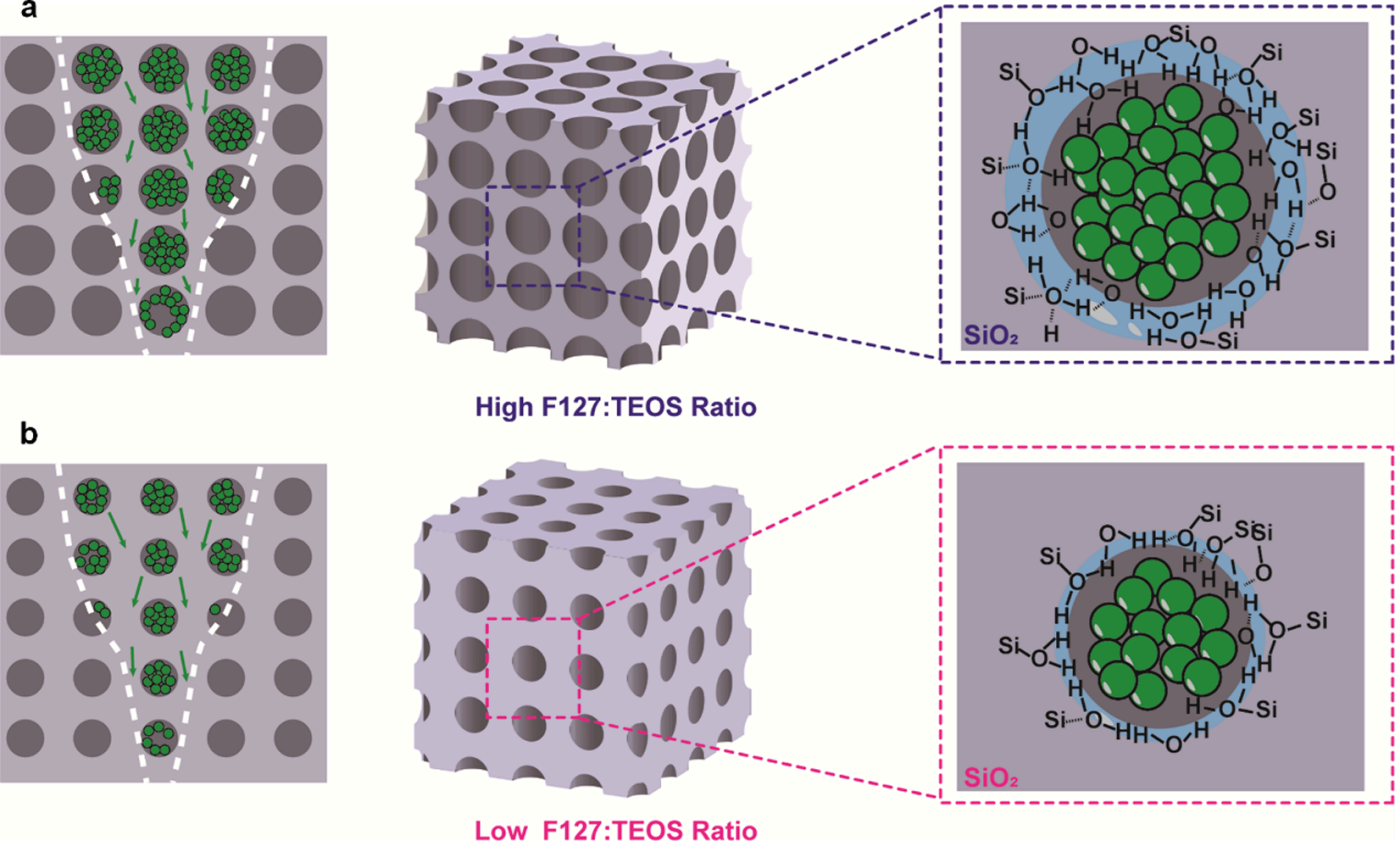
Illustration of an internal atom dynamics of devices with different porosity: (a) higher porosity and (b) lower porosity depicting the corresponding filament status during switching.

The migration of the Ag ions is also strongly influenced by the pore structures. Unlike solid film memristors where the conductive filaments are normally formed through the film defects and grain boundaries, the presence of mesoporous in our mSiO_2_ films was designed to facilitate the formation of filaments. It has been suggested that the interfacial energy and strain energy required for filament nucleation are significantly reduced due to the introduction of large surface area from pores in the mSiO_2_ layer.^25,34,39,54^ This provides low-energy pathways for the formation of multiple Ag filaments. This argument agrees well with our DC-IV characteristics (Figures 4 and 5) where memristors with high porosity operate at higher currents through the formation of stronger filaments, as illustrated in Figure 10. Similarly, when the memristors are stimulated by the same pulse signals, those with high porosity respond with higher PSCs (Figure 6). When the porosity is low, the migration of Ag ions in the film is limited, resulting in weaker filament(s) and lower current after the initial formation process. However, when subsequent stimulations are applied, substantial amount of Ag ions can still drift into the film. This results in significant growth of filament(s) and causes the large current change. On the other hand, for memristors with high porosity films, stronger filament(s) can be formed after the initial formation process. Although the consecutive stimulation pulses still generate Ag ions into the film, the growth of the filament is now less significant, resulting in smaller current changes as observed in Figure 7.

**Figure 11:**
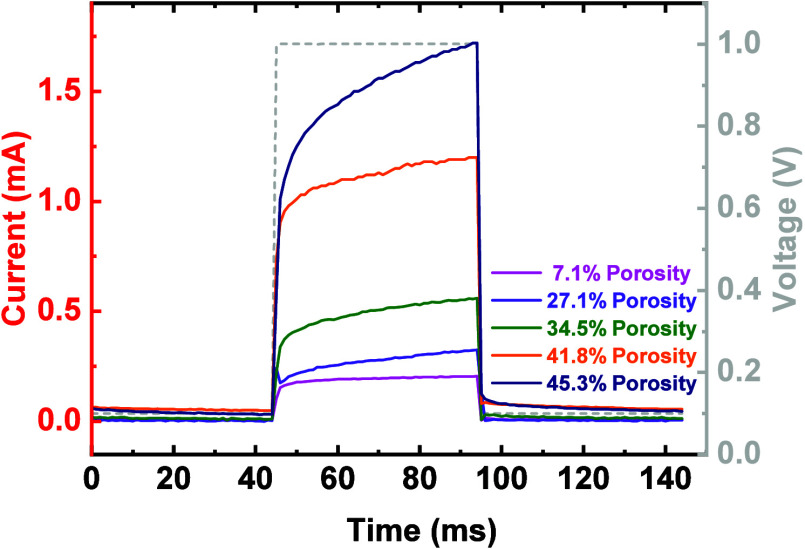
In-pulse measurement detecting current response under a 1 V, 50 ms duration pulse for mSiO_2_-based memristors with varying porosities.

To further verify this behavior, we monitored the current response of the memristors during the stimulation, which is shown in Figure 11. It can be observed that for low porosity memristors, the current increases at the start of the stimulus but saturated very soon at a relatively low level. This saturation implies that the further growth of the Ag filament is limited by (1) the counter-electrode reactions at the cathode interface and (2) the surface area of the pore wall. On the other hand, current in high porosity memristors (e.g., 45.3%) manifests a continuous increase toward the end of the stimulus at a much higher current level. This suggests the growth of the filaments continues in the pores with sufficient counter-electrode reaction resources and surface areas.

The removal of the stimulus leads to the spontaneous diffusion of Ag filaments, while the pore walls provide low-energy diffusion pathways. With Ag atoms dissolving alongside the pore walls, the memristor demonstrates short-term plasticity. However, the relaxation time is observed to be strongly dependent on the porosity of the film due to the various strengths of filaments obtained under the same stimulation. Such control over the dynamic performance of the filament is extremely beneficial for processing temporal signals with different time scales.

## 5 Conclusions

We synthesized mesoporous silica films through a sol–gel process with controllable porosities that can be used as functional electrolyte layers in memristor devices. The impact of the silica porosity on the memristor switching dynamics was systematically investigated. While all memristors demonstrate similar nonvolatile switching at large operating voltage and short-term plasticity at small operating voltage, the ones with higher silica film porosity feature higher operating currents, lower facilitation ratios, and longer relaxation times. Such control over the switching dynamics originates from the modulation of the hydrogen-bonded networks generated by the porous structure within the silica layer, which plays a crucial role in both the anodic oxidation and the ion migration processes during switching. The mesoporous silica layer provides a unique platform to regulate the ion dynamics in the diffusive memristor. The engineering of the porous structure opens new pathways to novel types of neuromorphic computing systems that can process temporal and sequential information.

## Supporting Information Available

Data and discussion on thin film GISAXS, refractive index, thin film thickness, AFM images, *I*–*V* characteristics of mesoporous silica-based memristors, endurance/retention characteristics, SCLC mechanism fitting, learning and forgetting rehearsal of mesoporous silica-based memristors, and XPS spectrum analysis (PDF)
