# Supplementary material for: Tunable Neuromorphic Switching Dynamics via Porosity Control in Mesoporous Silica Diffusive Memristors
Source: ACS Appl Mater Interfaces. 2024 Mar 18;16(13):16641–52. doi: 10.1021/acsami.3c19020 (PMC10995907; doi:10.1021/acsami.3c19020)
Supplement: Supplementary file 1 — am3c19020_si_001.pdf [file am3c19020_si_001.pdf]

Supporting Information

Tunable Neuromorphic Switching Dynamics via Porosity Control in Mesoporous Silica Diffusive Memristors

Tongjun Zhang[a], *Li Shao*[b], *Ayoub Jaafar*[a], *Ioannis Zeimpekis*[a], *Cornelis. H. de Groot*[a], *Philip N. Bartlett*[b], *Andrew L. Hector*[b], *and Ruomeng Huang*[a*]

[a]School of Electronics and Computer Science, University of Southampton, Southampton, United Kingdom

[b]School of Chemistry, University of Southampton, Southampton, United Kingdom

*Email: r.huang@soton.ac.uk

The mesostructured phases of the silica films were investigated by grazing-incidence small-angle X-ray scattering (GISAXS) and the results are presented in **Figure** S1. Due to increasing precursor F127:TEOS ratios, the GISAXS signals show various orders. To define the differential of GISAXS signals, we exported vertical profiles focusing on the peak shifts of as-deposited mesoporous silica samples. The vertical profile image is also plotted in **Figure** S1f. To classify our mesoporous silica structure, the transmitted and reflected Bragg reflections were calculated for each sample. The samples with 0.001 and 0.003 F127:TEOS ratios are

characterized as low-order structures because their intricacies are not likely to be captured. The sample with 0.005 F127:TEOS ratio was found to be a highly-ordered mesoporous silica film, the GISAXS pattern matches well with the orthorhombic *Fmmm* pore structure with most domains oriented with the (0 1 0) plane vertical to the substrate.[1] The lattice parameters are a=10.5 nm, b=8.5 nm and c=15 nm. The samples with 0.007 and 0.009 display a few Bragg peaks and two rings. It indicates that the mesostructures are less ordered and mesostructure domains orient in different directions. In both 0.007 and 0.009 samples, the Bragg peaks are horizontal to the Yoneda peak and vertical to the Yoneda peak, all matching well with the *Fmmm* mesostructure, with the (0 1 0) plane normal to the substrate. Moreover, the SEM images (**Figure** 2a-2e) show that samples with 0.005, 0.007 and 0.009 ratios have similar pore arrangements from the top view. It indicates that samples with 0.007 and 0.009 ratios have the same mesostructured as sample 0.005. However, because of less ordering, there are not enough Bragg peaks observed to simulate the lattice parameter values.

To investigate the spatial arrangement of the mesostructured phase, vertical line profiles were exported from the processed data through DPDAK software. As shown in **Figure** S1f, the q_y axis illustrates the intensity calculated from the Yoneda position. The vertical profiles show a broad peak at around 0.5 nm^{-1} position for all samples with different F127:TEOS ratios, where the broad peak shifted toward a lower position as the F127:TEOS ratio increased.[2-4]

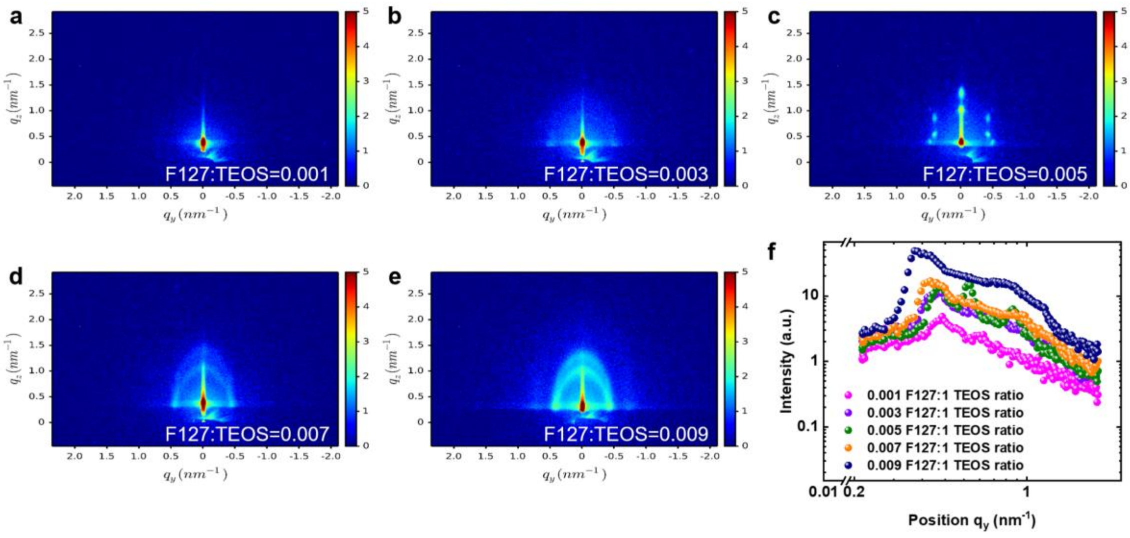

Figure S1 The GISAXS patterns of the mesoporous silica film with (a) 0.001, (b) 0.003, (c) 0.005, (d) 0.007, and (e) 0.009 F127 to TEOS ratio. (f) Vertical GISAXS profiles of the mesoporous silica films.

The refractive indices and film thickness were fitted using a Cauchy dispersion model. For comparison, the refractive indices of films with different F127:TEOS ratios and the silica film deposited by thermal oxidation are shown in **Figure** S2a. For all samples, the refractive index decreases as the incident wavelength increases from 200 nm to 1700 nm. The refractive indices of mesoporous silica layers are confirmed to be lower than those of the thermally oxidized samples. The thicknesses of the as-deposited mesoporous silica layers are plotted in **Figure** S2b. It was observed that the film thickness increases with increasing F127:TEOS ratio.

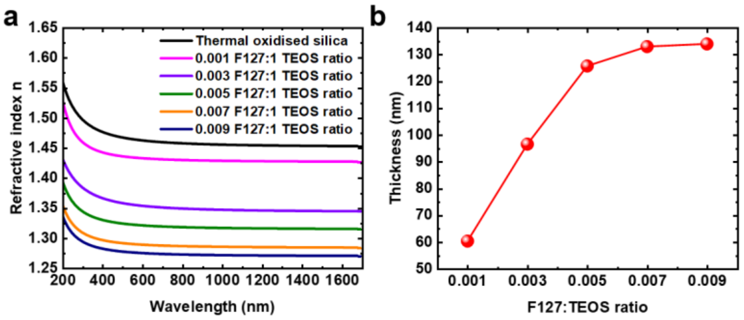

Figure S2 (a) Refractive index change collected over 200–1700nm wavelength range for mesoporous silica films with different F127:TEOS ratios on the silicon substrate; (b). The thickness versus F127:TEOS ratio in mesoporous silica layers.

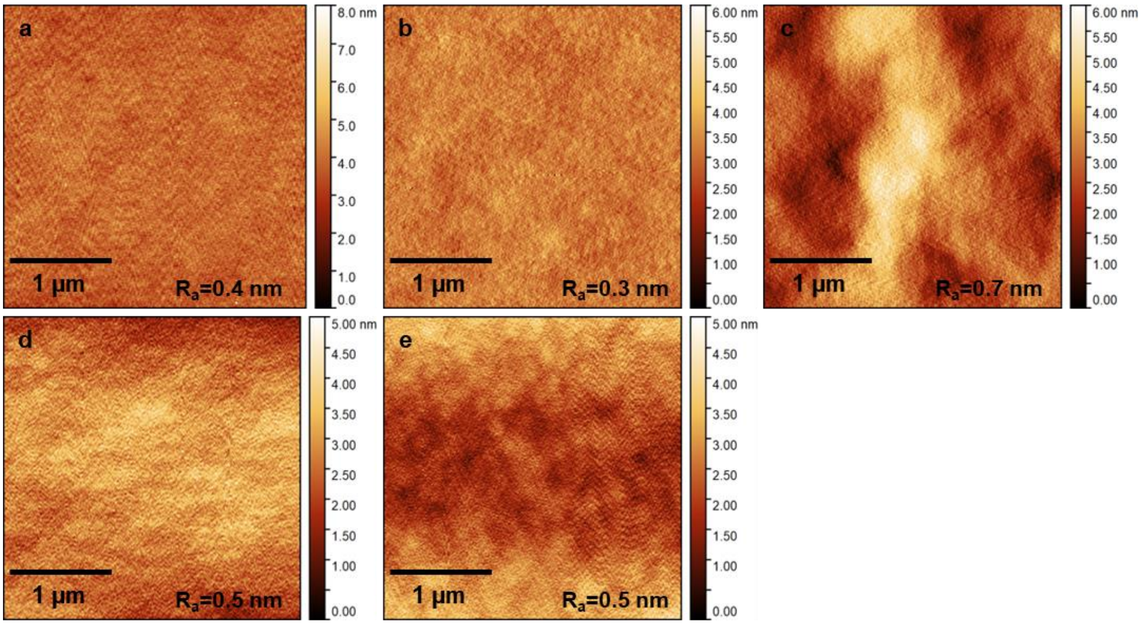

Figure S3 Atomic force microscope images showing low surface average roughness (R_a) of samples with different porosity: (a) 7.1%, (b) 27.1%, (c) 34.5%, (d) 41.8%, (e) 45.3%.

The retentions of devices with different porosity were exhibited in **Figure** S4. The resistance states were read at 0.1 V, and the reading current in LRS and HRS exhibited excellent stability after 10^3 s, confirming the non-volatility of the memristors. This implies formation of strong filaments in all memristors. Under this condition, the impact of porosity on the retention is not significant. **Figure** S5 depicts the endurance of the non-volatile switching behaviors of our memristors where repeatable switching can be observed in all memristors. It is worth noting that the non-volatile endurance performance of our memristors were not optimized.

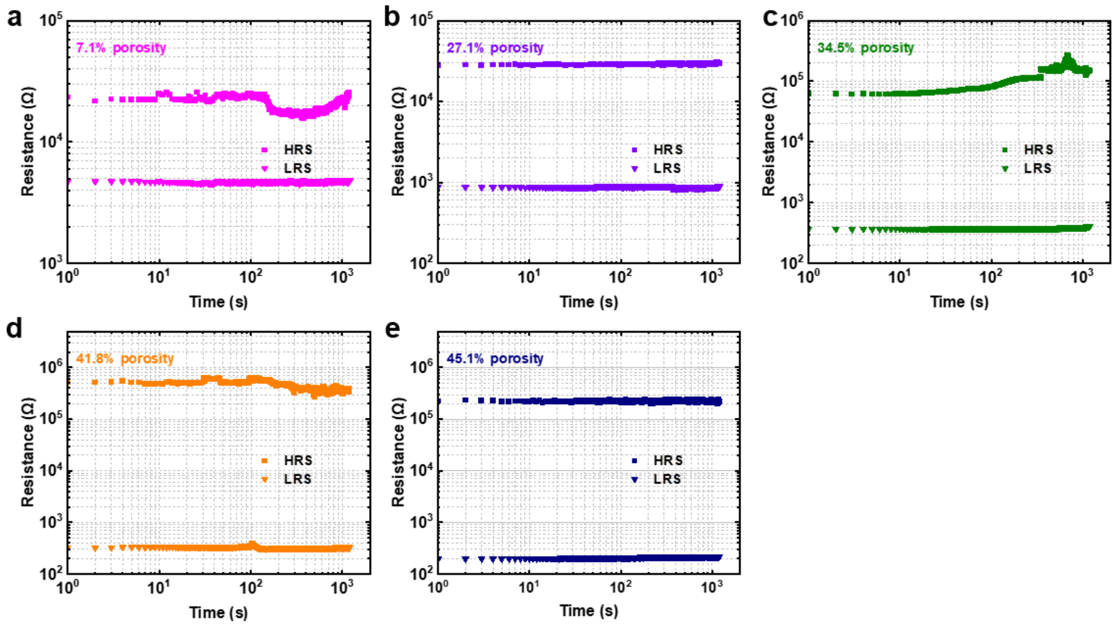

Figure S4 Retention characteristics for non-volatile switching behavior of memristor with different porosity: (a) 7.1%, (b) 27.1%, (c) 34.5%, (d) 41.8%, (e) 45.3%.

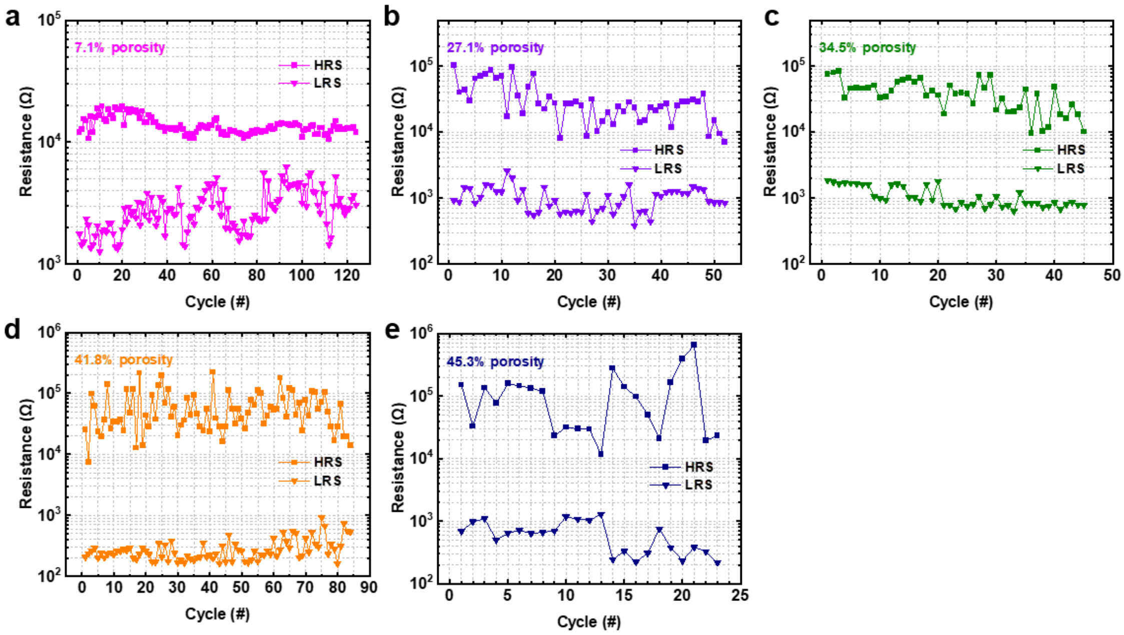

Figure S5 Endurance characteristics for non-volatile switching behavior of memristor with different porosity: (a) 7.1%, (b) 27.1%, (c) 34.5%, (d) 41.8%, (e) 45.3%.

To demonstrate the variation of switching voltages, the distribution of programming voltages for all devices is illustrated in **Figure** S6. It can be observed that all devices have similar set and reset voltages at around +1 V and -2 V, respectively.

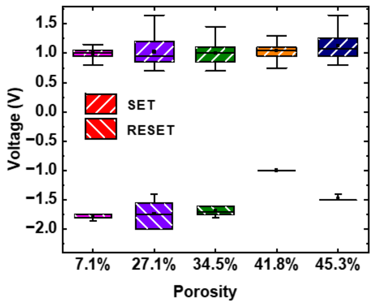

Figure S6 Distribution of the programming voltages for the $mSiO_2$-based memristors with different porosities.

Figure S7 plots the I-V characteristic of all memristor devices in the log-log scale. Taking the device with 45.3% porosity as an example, initially from 0 V to 0.15 V at HRS, the device shows a linear dependence of current with applied voltage. An Ohmic conduction mechanism is obtained, which arises from thermally generated charge carriers. At higher applied voltages ($0.2 \leq V \leq 0.9$ V), the slope changes to approximately 2($I \propto V^2$), and the current exhibits the voltage square dependence, which can be attributed to the trap-controlled space charge limited current.

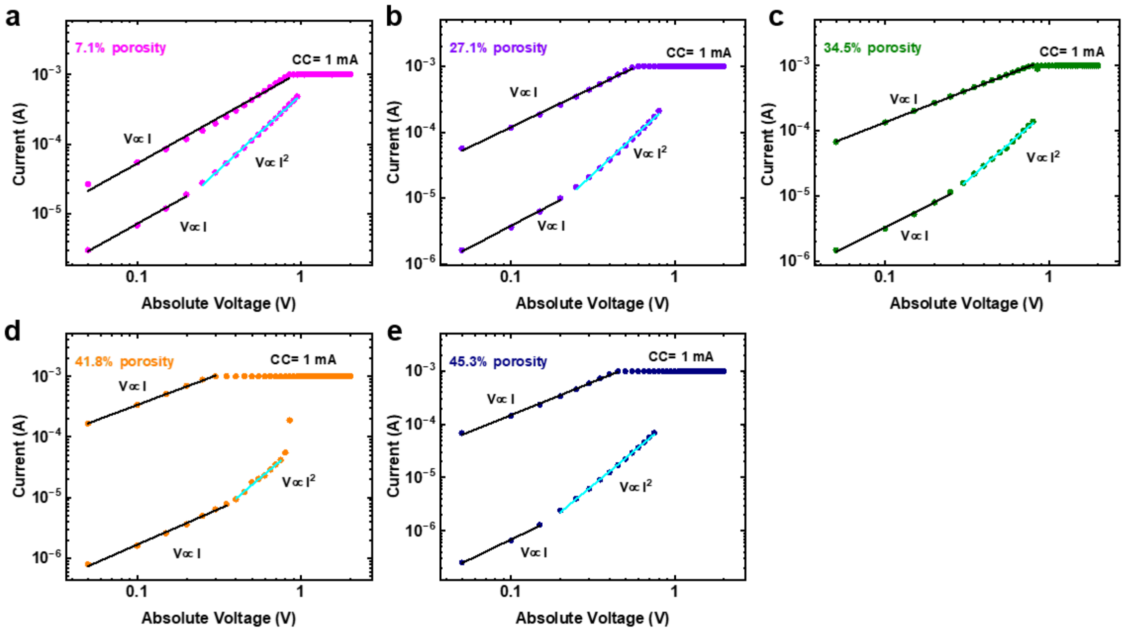

Figure S7 The I−V curves fitting with SCLC modes in different voltage regions of memristor with different porosity: (a) 7.1%, (b) 27.1%, (c) 34.5%, (d) 41.8%, (e) 45.3%.

Figure S8 illustrates the relationship between current values and consecutive cycles in the HRS of the device, with current values recorded at 0.5 V. All HRS display a similar trend with LRS, where the current state increases gradually with each repeating DC cycle in all memristors. In addition, the current states also increase with increasing film porosity.

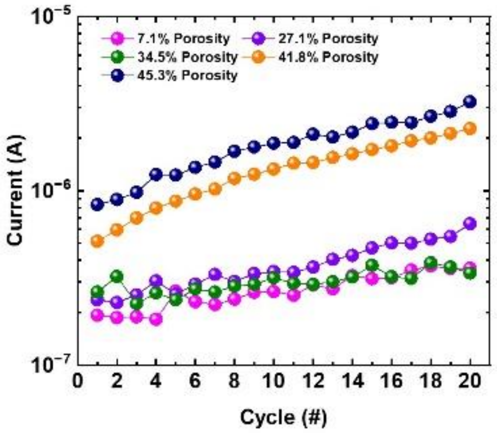

Figure S8 Current changes versus consecutive cycles of the HRS of the device. The current values were read at 0.5 V.

6 groups of pulses were applied onto the 5 memristors. Each group consists of 50 pulses with 1 V potential, 50 ms duration, and 50 ms interval. The PSC after the individual pulse is recorded. Then the external pulse signals are removed and the current states for each sample are still measured at 0.1 V. The measurement results are shown in **Figure** S9. The red curves in **Figure** S9a to **Figure** S9e illustrate the pulse-induced potentiation process and the blue curves represent the depression process. Under pulse stimulations, the current of devices gradually increases. This is followed by spontaneous current decay after the withdrawal of the stimulation. All memristor devices show similar current enhancement and depression behaviors. There is still an overall memristor conductance increase despite obvious current relaxation. The retention loss shows a significant difference among these five samples, indicating the dissolution process of filament structure in different mesoporous silica layers is various.

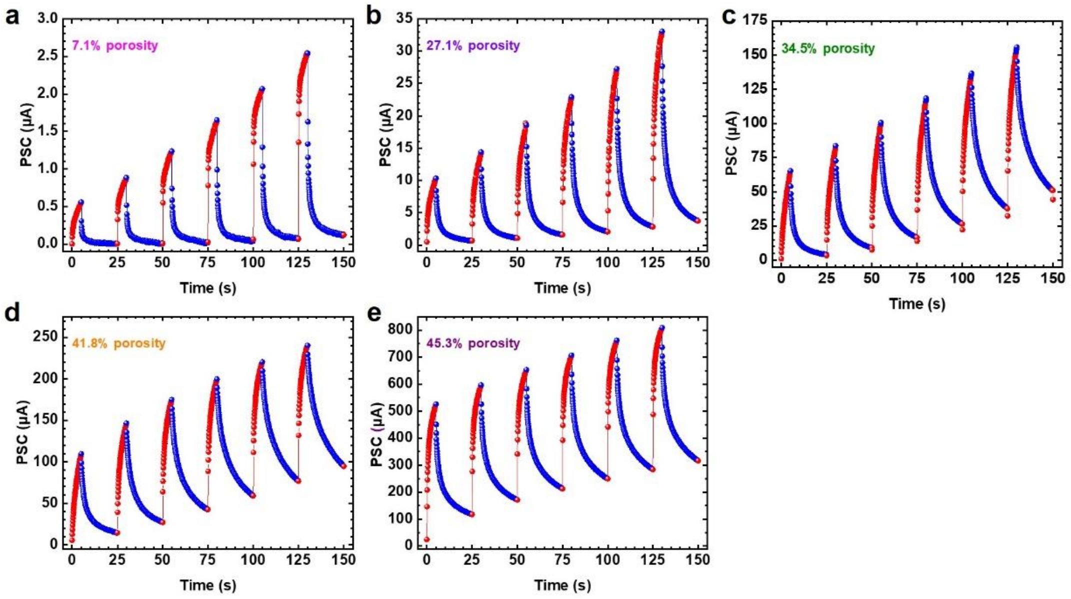

Figure S9. Current response at consecutive switching with fixed pulses signals under learning and forgetting rehearsal of 6 cycles for mSiO_2 samples with different porosity:(a) 7.1%, (b) 27.1%, (c) 34.5%, (d) 41.8%, (e) 45.3%.

Table S1 provides a summary of the memristive capabilities observed in different SiO_2 and porous material based memristors.

Table S1 An overview of the memristive capabilities observed in different SiO_2 and porous material based memristors.

Ref	Film	Device structure	Programming voltages	Deposition Method	Relaxation window	Pore control
5	Dense	TaN/SiO_2/N^+Si	4 V/5 V	CVD	n/a	n/a
6	Dense	Ag/SiO_x/SiO_y/TiN	±0.5 V	sputtering	n/a	n/a
7	Dense	Ag/Ag-doped $SrTiO_3$/SiO_2/p^{++}-Si	±5 V	sputtering	n/a	n/a
8	Dense	Ag/Ag-doped $SrTiO_3$/SiO_2/p^{++}-Si	5 V, –4.5 V	PVD	n/a	n/a
9	Dense	Pt/SiO_2:Ag/TiO_2/p^{++}-Si	±3.5 V	sputtering	n/a	n/a
10	Porous	Al/mesoporous silica−titania/Pt	±5 V, ±7 V&±10 V	Evaporation-induced self-	n/a	n/a

				assembly synthesis		
11	Porous	Pt/PLiCoO$_2$/porous SiO$_x$ with quantum dots/Si	±5 V	Electrochemical etch and thermal oxidation	75–350 ms	n/a
12	Porous	Pt/Multilayer graphene /nanoporous Ta$_2$O$_{5-x}$ /Ta/Pt	±8 V	Electrochemical anodizing	n/a	porosity
13	Porous	Pt/TaO$_y$/nanoporous TaO$_x$/Ta	±8 V	Oxidation and anodization	n/a	n/a
14	Porous	Ag/Ag$_2$S/mesoporous silica/ITO	2 V	Stöber solution growth	0.02 s & 4 s	pore orientation
15	Porous	Ag/porous SiO$_x$/Si	±5 V	CVD	–	–
**This work**	Porous	Ag/mesoporous silica/ TiN	±2 V	Solution-based dip coating	0.3 s to 27 s	porosity

Figure S10 presents the gradual change of the envelope peak position with changing mSiO$_2$ film porosity. It can be observed the peak shifts towards higher energy with increasing porosity, implying more high-energy bonds (hydroxyl groups) are available in the film.

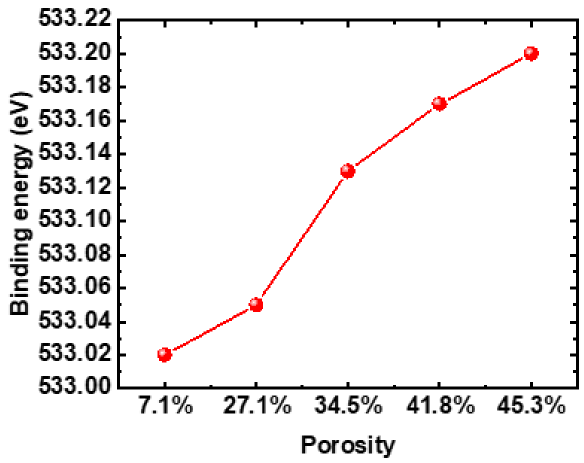

Figure S10. XPS envelope peak position as a function of film porosity.

Reference

[1] Shao, L.; Moehl, G. E.; Huang, R.; Hector, A. L. Fractal-Like Gold Nanonetworks Formed by Templated Electrodeposition Through 3D-Mesoporous Silica Films. *RSC Adv* **2023**, *13*, 32660-32671. DOI: 10.1039/d3ra06588j

[2] Yang, J.; Zhai, Y.; Deng, Y.; Gu, D.; Li, Q.; Wu, Q.; Huang, Y.; Tu, B.; Zhao, D. Direct Triblock-Copolymer-Templating Synthesis of Ordered Nitrogen-Containing Mesoporous Polymers. *J Colloid Interface Sci* **2010**, *342*, 579-85. DOI: 10.1016/j.jcis.2009.10.037

[3] Chen, D.; Li, Z.; Wan, Y.; Tu, X.; Shi, Y.; Chen, Z.; Shen, W.; Yu, C.; Tu, B.; Zhao, D. Anionic Surfactant Induced Mesophase Transformation to Synthesize Highly Ordered Large-Pore Mesoporous Silica Structures. *Journal of Materials Chemistry* **2006**, *16*. DOI: 10.1039/b517975k

[4] Jiang, Z. GIXSGUI: A MATLAB Toolbox for Grazing-Incidence X-ray Scattering Data Visualization and Reduction, and Indexing of Buried Three-Dimensional Periodic Nanostructured Films. *Journal of Applied Crystallography* **2015**, *48*, 917-926. DOI: doi:10.1107/S1600576715004434

[5] Fowler, B. W.; Chang, Y.-F.; Zhou, F.; Wang, Y.; Chen, P.-Y.; Xue, F.; Chen, Y.-T.; Bringhurst, B.; Pozder, S.; Lee, J. C. Electroforming and Resistive Switching in Silicon Dioxide Resistive Memory Devices. *RSC Advances* **2015**, *5*, 21215-21236. DOI: 10.1039/c4ra16078a

[6] Bousoulas, P.; Panagopoulou, M.; Boukos, N.; Tsoukalas, D. Emulating Artificial Neuron and Synaptic Properties with SiO_2-Based Memristive Devices by Tuning Threshold and Bipolar Switching Effects. *Journal of Physics D: Applied Physics* **2021**, *54*. DOI: 10.1088/1361-6463/abea3b

[7] Ilyas, N.; Wang, J.; Li, C.; Fu, H.; Li, D.; Jiang, X.; Gu, D.; Jiang, Y.; Li, W. Controllable Resistive Switching of STO:Ag/SiO_2-Based Memristor Synapse for Neuromorphic Computing. *Journal of Materials Science & Technology* **2022**, *97*, 254-263. DOI: 10.1016/j.jmst.2021.04.071

[8] Ilyas, N.; Li, C.; Wang, J.; Jiang, X.; Fu, H.; Liu, F.; Gu, D.; Jiang, Y.; Li, W. A Modified SiO_2-Based Memristor with Reliable Switching and Multifunctional Synaptic Behaviors. *J Phys Chem Lett* **2022**, *13*, 884-893. DOI: 10.1021/acs.jpclett.1c03912

[9] Li, D.; Ilyas, N.; Li, C.; Jiang, X.; Jiang, Y.; Li, W. Synaptic Learning and Memory Functions in SiO_2:Ag/TiO_2 Based Memristor Devices. *Journal of Physics D: Applied Physics* **2020**, *53*. DOI: 10.1088/1361-6463/ab70c9

[10] Jung, H.; Kim, Y. H.; Kim, J.; Yoon, T. S.; Kang, C. J.; Yoon, S.; Lee, H. H. Analog Memristive Characteristics of Mesoporous Silica-Titania Nanocomposite Device Concurrent with Selection Diode Property. *ACS Appl Mater Interfaces* **2019**, *11*, 36807-36816. DOI: 10.1021/acsami.9b09135

[11] Gao, Q.; Huang, J.; Gao, J.; Geng, X.; Ji, Y.; Li, H.; Wang, G.; Liang, B.; Wang, M.; Xiao, Z.; et al. Tunable Plasticity in Functionalized Honeycomb Synaptic Memristor for Neurocomputing. *Materials Today Physics* **2023**, *30*. DOI: 10.1016/j.mtphys.2022.100947

[12] Kwon, S.; Kim, T. W.; Jang, S.; Lee, J. H.; Kim, N. D.; Ji, Y.; Lee, C. H.; Tour, J. M.; Wang, G. Structurally Engineered Nanoporous Ta_2O_{5-x} Selector-Less Memristor for High Uniformity and Low Power Consumption. *ACS Appl Mater Interfaces* **2017**, *9*, 34015-34023. DOI: 10.1021/acsami.7b06918

[13] Choi, S.; Jang, S.; Moon, J.-H.; Kim, J. C.; Jeong, H. Y.; Jang, P.; Lee, K.-J.; Wang, G. A Self-rectifying TaO_y/Nanoporous TaO_x Memristor Synaptic Array for Learning and Energy-Efficient Neuromorphic Systems. *NPG Asia Materials* **2018**, *10*, 1097-1106. DOI: 10.1038/s41427-018-0101-y

[14] Li, B.; Liu, Y.; Wan, C.; Liu, Z.; Wang, M.; Qi, D.; Yu, J.; Cai, P.; Xiao, M.; Zeng, Y.; et al. Mediating Short-Term Plasticity in an Artificial Memristive Synapse by the Orientation of Silica Mesopores. *Adv Mater* **2018**, *30*, e1706395. DOI: 10.1002/adma.201706395

[15] Li, H.; Gao, Q.; Gao, J.; Huang, J.; Geng, X.; Wang, G.; Liang, B.; Li, X.; Wang, M.; Xiao, Z.; et al. Controllability of the Conductive Filament in Porous SiO_x Memristors by Humidity-Mediated Silver Ion Migration. *ACS Appl Mater Interfaces* **2023**, *15*, 46449-46459. DOI: 10.1021/acsami.3c07179
